# An Unusual Presentation of a Primary Chondrosarcoma of the Cranial Vault

**DOI:** 10.7759/cureus.60398

**Published:** 2024-05-16

**Authors:** Zeus Mala, Omar Ksiks, Hajar Hamadi, Lamia Benantar, Khalid Aniba

**Affiliations:** 1 Neurological Surgery, Ibn Tofail Hospital, Mohammed VIth University Hospital, Marrakech, MAR

**Keywords:** cartilaginous tumour, cranial vault, chondrosarcoma, calvarium, primary chondrosarcoma

## Abstract

Chondrosarcomas are malignant cartilaginous tumors that usually affect the pelvic bone and long bones. Primary chondrosarcomas of the skull are rare, with the cranial vault being an even more unusual localization. We report a case of a 75-year-old man presenting with headaches and outgrowth of the parietal scalp. CT scan of the head showed an extracranial cystic well-rounded mass originating at the parietal suture and eroding through the adjacent parietal bone. The patient underwent an en bloc surgical resection of the mass, and histological examination confirmed a grade I chondrosarcoma.

## Introduction

Chondrosarcomas (CSs) are slow-growing malignant cartilaginous tumors, usually affecting the pelvic bone and long bones metaphysis and diaphysis [1,2]. The skull remains an extremely rare localization and CS from extracranial sources rarely metastasize to the brain [3,4]. Primitive chondrosarcomas (PCS) represent 0.16% of intracranial neoplasms [3,5]. The base of the cranium is the most frequent intracranial localization, specifically the petrous and sphenoid bones [2]. The cranial vault remains less affected by these tumors [2].

Intracranial CSs are a heterogeneous group of tumors with various morphological features and clinical symptoms. They manifest with signs and symptoms of local invasion and mass effect on the surrounding structures as a result, the most frequent presentation is intracranial hypertension [2,3,5]. This wide range of symptoms coupled with unspecific imaging results rarely allows for a preoperative diagnosis [5]. As a result, surgery with a histological analysis is mandatory to confirm the diagnosis [2,5,6]. We report a rare case of primary cranial vault chondrosarcoma with an unusual presentation.

## Case presentation

The patient was a 75-year-old man with no significant personal or family medical history (specifically no prior history of surgery or tumors). He presented to our department of neurosurgery at Ibn Tofail Hospital, Mohamed 6 University Hospital, in Marrakech with a four-month history of progressively worsening headaches associated with a growing mass of the midline scalp. Physical examination revealed a conscious well well-oriented elderly man with no neurological deficit. Scalp examination showed a well-rounded regular and smooth mass of the midline parietal scalp, soft in consistency and painful on palpation, with no apparent skin modifications measuring approximately 3 cm in diameter. Given the location and nature of the lesion, we initially thought of a lipoma or an epidermoid cyst.

**Figure 1:**
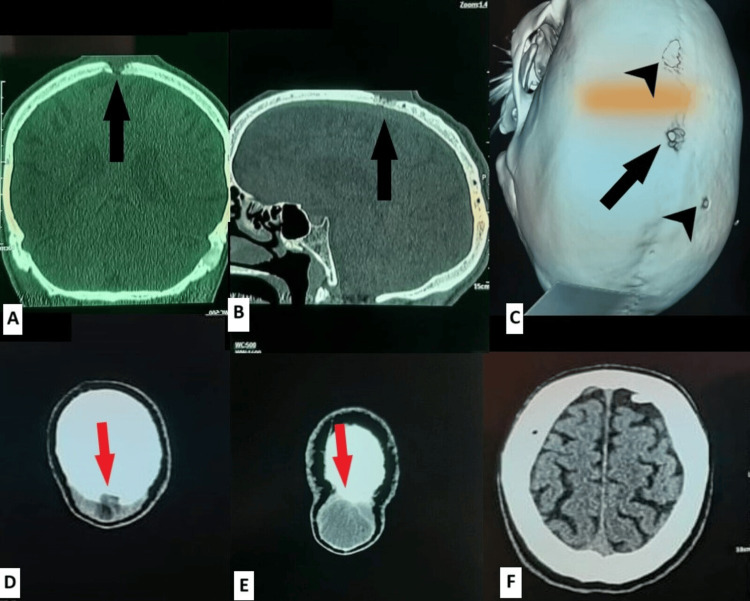
Preoperative CT scan images showing the midline mass. (A) CT scan of the head in coronal planes bone window showing a midline bone defect (black arrow). (B) CT scan of the head in sagittal planes bone window showing bone erosion in the parietal bone(black arrow). (C) 3D reconstruction of the preoperative CT scan showing a rounded bone defect at the parietal suture (black arrow) as well as bone defects (black arrowhead) at the left parietal bone and the right frontal bone. (D) and (E) CT scan of the head in axial planes parenchymal window showing a bulging cystic mass with extracranial and intracranial components (red arrow). (F) CT scan of the head in the axial planes parenchymal window showing the intact brain parenchyma with apparent signs of invasion or mass effect from the intracranial portion of the mass.

The patient underwent a CT scan of the head showing a midline parietal cystic lesion of the scalp measuring 33 mm x 36 mm eroding through the underlying bone, with multiple lesions of the cranial vault individualized on 3D reconstruction (Figure 1). The results of the imaging were suggestive of a dermoid cyst. Surgical treatment was proposed to the patient, and after a clear explanation of his condition, evolution, and potential surgical risks, informed consent was obtained for the procedure.

**Figure 2:**
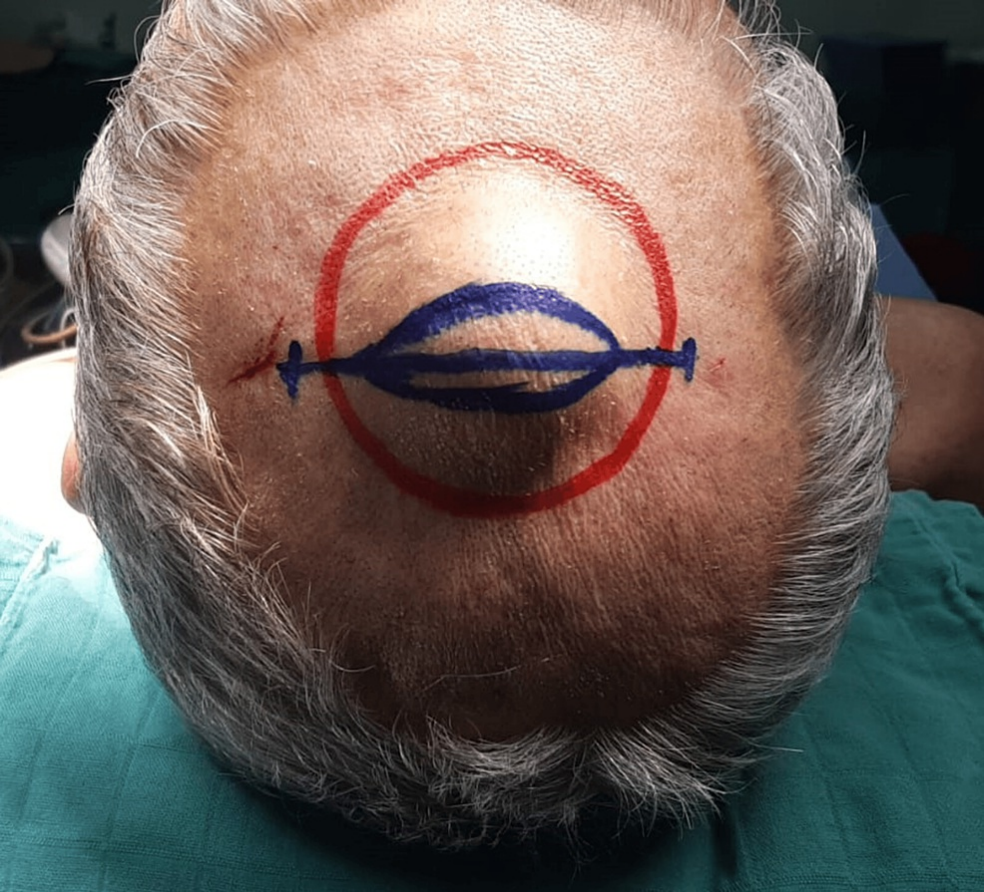
intraoperative image of the midline mass showing the head position as well as the incision line. The intraoperative image showing a well-rounded bulging mass at the interparietal suture with no apparent skin changes, measuring approximately 3 cm in diameter. The image shows the incision line chosen to better expose the lesion and perform an en bloc resection.

Surgical resection was carried out under general anesthesia. A horizontal linear incision was made followed by careful dissection making sure not to pop the cystic mass (Figure 2).

**Figure 3:**
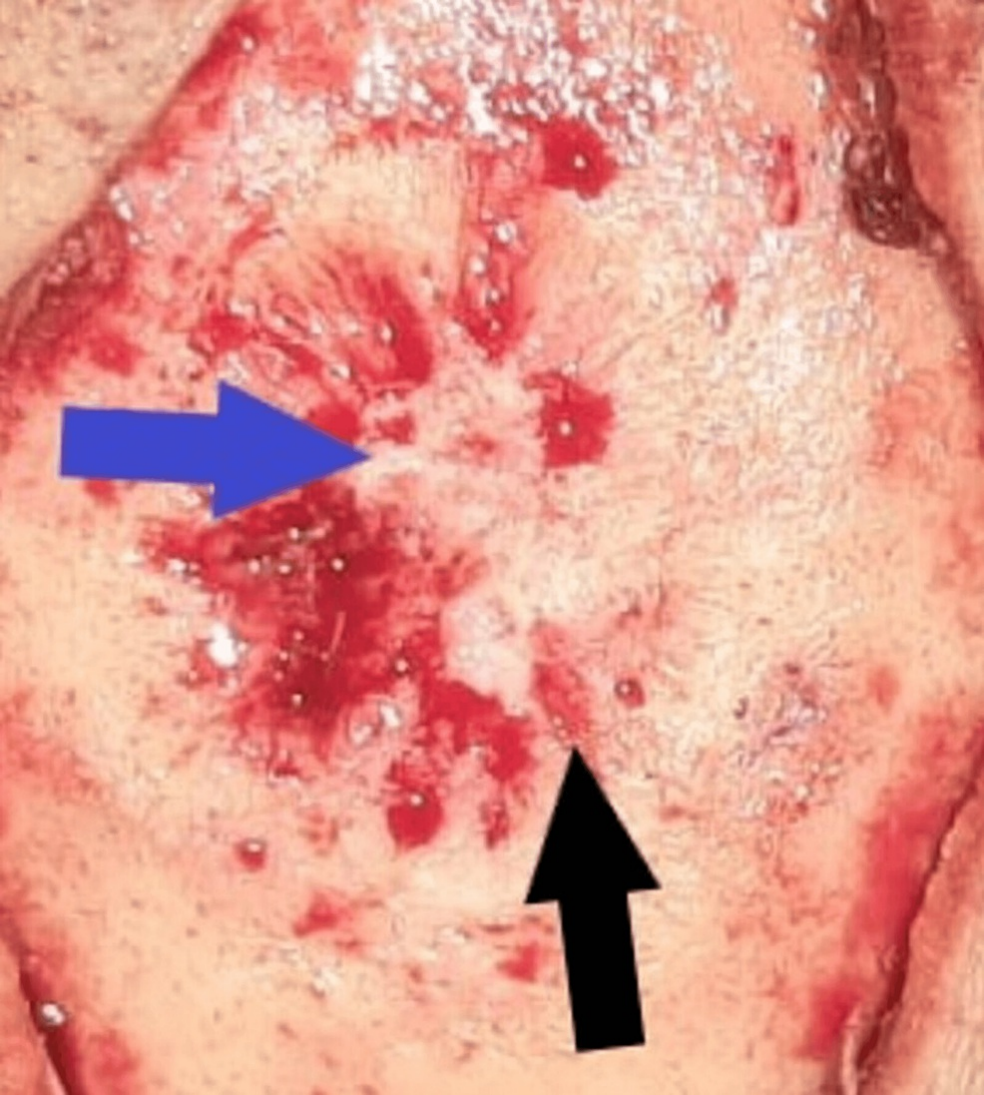
Intraoperative image of the print in the underlying parietal bone after en bloc resection of the mass. The underlying bone was infiltrated and friable with a depression (black arrow) left by the gelatinous mass after resection. This depression was centered with a small defect (blue arrow). The underlying dura matter showed no sign of infiltration.

Perioperatively, the lesion was subgaleal, well-rounded, and encapsulated, easily detached from the surrounding tissue but adherent to the underlying bone. En bloc excision of the mass revealed a depression on the parietal bone with a small defect (Figure 3). The underlying infiltrated bone was friable and was removed carefully, making sure not to open the dura mater or wound the superior sagittal sinus. Both the mass and the excised bone fragments were sent for pathological examination.

**Figure 4:**
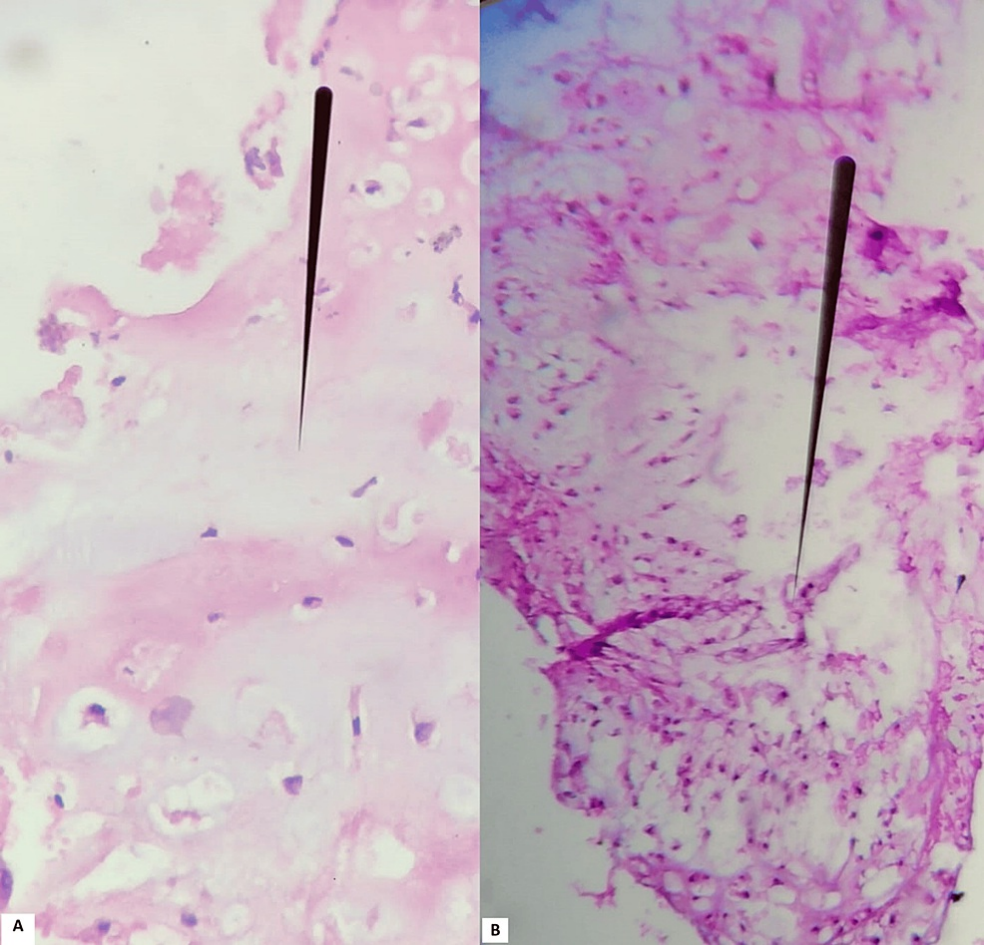
Images of the histopathological examination results. (A) HEx40 atypical proliferation of cartilaginous cells with well-differentiated cartilaginous cells. (B) HEx10 atypical cartilaginous cell proliferation.

On histopathological examination, the cystic mass and infiltrated bone fragments showed an atypical proliferation of cartilaginous cells with well differentiated cartilaginous cells, which was consistent with a well-differentiated chondrosarcoma (World Health Organization grade I chondrosarcoma) (Figure 4). Postoperative follow-up was uneventful. The patient underwent a workup to identify secondary localizations of the chondrosarcoma, which was normal, and he was discharged with a referral to the oncology department for postoperative radiotherapy.

## Discussion

Intracranial CSs are rare subtypes of CSs first described in 1899 by Mott [4,5]. As an entity, CSs are the second most common primary malignant cartilaginous tumors and the third most common primary malignant neoplasms of bones [2,7]. Typically, they occur in middle-aged individuals with no gender preferences, although a slight male predominance has been reported in more recent reviews [4,5]. Intracranial CSs usually arise in the paraclival region developing from sphenopetrosal synchondrosis and petroclival synchondrosis, but in some rare cases, they can be found in the cerebral meninges, parenchyma, or vascular plexus [1,2,8]. Intracranial PCSs are not unusual, and most cases are sporadic with no identifiable risk factor [1,2]. However, CS could be associated with some rare skeletal disorders, including Ollier disease, Maffucci syndrome, Paget’s disease, and osteochondromas [1,2,6]. Histologically, they are classified into three subtypes according to the World Health Organization (WHO 2020): well-differentiated (grade I), intermediate or myxoid (grade II), and undifferentiated or mesenchymal (grade III) [2,5,6]. Grade III represents the most common subtype arising above the skull base with a predilection for young adults aged 10 to 30 years [2]. This grading system represents the main prognostic factor [6,7].

The pathogenesis explaining the occurrence of these tumors in the skull is multiple. On an embryological level, the bones of the cranial vault predominantly develop through intramembranous ossification. However, endochondral ossification is also involved in the development of several sites, including a large part of the petrous portion of the temporal bone, the areas of the petro-occipital, spheno-occipital, and spheno-petrosal synchondroses. As a result, it is believed that intracranial CS might develop from the chondrocytes within the rest of the endochondral cartilage that may be present in these areas [1,2,9,10].

The clinical presentation was due to the direct compression on surrounding structures. In the case of CS of the skull base, the most common presentation is headaches and diplopia as a result of cranial nerves and neurovascular structures compression [5]. In our case, the patient presented with headaches and a slowly growing mass. The presence of headache was consistent with previously described cases of intracranial PCS and represented the second most common symptom after diplopia [1,2,7]. Unlike previously described cases, the presence of a bulging mass was particular to our case since the growth of the tumor was extracranial and originated from the junction of the parietal bones at the interparietal sagittal suture.

CT scan of the head usually shows an isodense or hyperdense mass with a heterogeneous contrast uptake and varying degrees of calcification [2,4]. In T1 MRI, the tumor is usually iso-/hypo-intense, and in T2 MRI sequences, it is mostly mixed density with no or mild brain edema [2,4,11]. In contrast-enhanced images, the tumor enhancement is poor and heterogeneous [11]. En bloc surgical excision is the preferred treatment for CS [2,11,12]. In skull base CS, the choice of the surgical approach depends on the tumor’s bony epicenter and extension and aims at safely exposing the mass and the surrounding vascular and nervous structures [1,4,5]. After careful planning, the controversy remains as authors argued for the benefits of gross total resection versus tumor reduction in skull base CS [1,4,5,11]. Furthermore, multiple studies suggest that surgery alone is not sufficient as the results showed no significant statistical difference between total or partial resection and that surgery was associated with a recurrence risk of 44% over five years, which can be reduced by adjuvant radiotherapy to merely 9% [2,4-6,8,12]. Although CSs are usually radioresistant due to their slow growth patterns, the effectiveness of post-resection radiotherapy in high-grade subtypes has changed the prognosis and the overall survival of these patients [4,5]. The use of carbon-ion-based radiotherapy has also been described in the management of CS. However, due to its limited availability, the benefits and risks are not well understood [5,8]. Proton beam radiotherapy in the treatment of CS has been investigated by many studies as a way of providing better local control in post-surgery tumor residues or unresectable CS [11].

The prognosis of intracranial CS is determined by several factors, with the major factor being the histological grade and type, but other factors such as tumor extent, quality of the surgical resection, and the use of postoperative adjuvant radiotherapy have also been described [2,5]. Bloch et al. established the five-year survival for each grade: grade I at 95%, grade II at 90%, and grade III at 75% [2,5,6]. However, other authors linked grade III CS with a lower survival rate compared to the aforementioned study (43% vs. 75%) [2]. In our case, the patient benefited from en bloc resection of the extracranial mass as well as the infiltrated adjacent bone and was referred to the oncology department to begin adjuvant radiotherapy, with no signs of recurrence during the three-month, six-month, and one-year follow-up.

## Conclusions

Primary CSs of the cranial vault are particularly rare malignant cartilaginous tumors. The diagnosis of these tumors is challenging since both the clinical and radiological aspects are nonspecific. Surgical debulking coupled with adjuvant radiotherapy shows better results with better local tumor control, a lower recurrence rate, and an improved survival rate. By sharing our case, we report an unusual presentation of a cranial vault location, adding it to the multitude of previously described cases and considering it as a potential differential diagnosis for outgrowth or bulging masses of the skull.

## References

[1] Konovalov A, Shekhtman O, Shekhtman AP, Bezborodova T (2020). Chondrosarcoma of the skull base: a case study and literature review. Cureus.

[2] Mishra K, Sharma S, Purohit DK, Jindal A (2021). Chondrosarcoma of cranial vault: case report and review of literature. Iran J Neurosurg.

[3] Liu H, Li Z, Xue Y, Zhao T, Wu Y (2023). A multicenter retrospective analysis of clinical outcomes of intracranial chondrosarcoma in 26 patients. Sci Rep.

[4] Chi J, Zhang M, Kang J (2016). Classical intracranial chondrosarcoma: A case report. Oncol Lett.

[5] Palmisciano P, Haider AS, Sabahi M (2021). Primary skull base chondrosarcomas: a systematic review. Cancers (Basel).

[6] Bloch OG, Jian BJ, Yang I, Han SJ, Aranda D, Ahn BJ, Parsa AT (2009). A systematic review of intracranial chondrosarcoma and survival. J Clin Neurosci.

[7] Smolle E, Mokry M, Haybaeck J (2015). Rare case of a primary intracranial chondrosarcoma. Anticancer Res.

[8] Lu VM, O'Connor KP, Mahajan A, Carlson ML, Van Gompel JJ (2020). Carbon ion radiotherapy for skull base chordomas and chondrosarcomas: a systematic review and meta-analysis of local control, survival, and toxicity outcomes. J Neurooncol.

[9] Gilbert SF (2017). Developmental biology, the stem cell of biological disciplines. PLoS Biol.

[10] Lau DP, Wharton SB, Antoun NM, Bottrill ID, Moffat DA (1997). Chondrosarcoma of the petrous apex. Dilemmas in diagnosis and treatment. J Laryngol Otol.

[11] Moin H, Sourani A, Sabouri M (2021). Large Parafalcine chondroma presenting with seizure: a case report. SN Compr. Clin. Med.

[12] Simon F, Feuvret L, Bresson D (2018). Surgery and protontherapy in Grade I and II skull base chondrosarcoma: a comparative retrospective study. PLoS One.

